# Protective role of lipoic acid in the prevention of oxidative stress caused by cadmium in the vascular endothelium of rats

**DOI:** 10.1192/j.eurpsy.2021.1905

**Published:** 2021-08-13

**Authors:** J. Jovanovic Mirkovic, C. Alexopoulos, Z. Jurinjak, M. Jerkan, N. Medojevic

**Affiliations:** 1 Health Studies, The Academy of Applied Preschool Teaching and Health Studies, cuprija, Serbia; 2 Physicals Medicine, Health center Nis, nis, Serbia; 3 Urology, Clinical Center Nis, nis, Serbia

**Keywords:** cadmium, blood vessel, antioxidant, oxidative stress, alpha-lipoic acid

## Abstract

**Introduction:**

Acute and chronic exposure to cadmium (Cd), due to its increased use and application in the industry, can result in the development of atherosclerosis, the occurrence of cardiomyopathy, cerebrovascular stroke, as well as carcinogenesis in some organs. The target for cadmium action is thought to be the vascular endothelium.

**Objectives:**

The aim of this study is to investigate the occurrence of oxidative stress on blood vessel endothelium induced by subacute administration of cadmium, as well as the protective power of alpha-lipoic acid (α-LA) supplementation on the Wistar strain albino rat model system.

**Methods:**

After anesthesia of rats in the vivarium of the Scientific Research Center for Biomedicine, Faculty of Medicine in Niš, blood was collected by cardiac puncture and sent to the Clinic of Nephrology, Clinical Center in Niš, Serbia for determination of hematological parameters.

**Results:**

According to the results of this study, it can be seen that the number of granulocytes is reduced due to cadmium intoxication, which is probably induced by the migration of neutrophils into tissues. The number of lymphocytes was increased due to subacute cadmium intoxication compared to the control group of animals. The positive efficacy of α-LA supplements in combating the adverse effects of cadmium on blood vessels is also confirmed.

**Conclusions:**

Cadmium administration is thought to cause a systemic inflammatory reaction due to the formation of free radicals in the blood vessel endothelium. Administration of α-LA supplement confirms that it can be used as an antioxidant in the clinical management of many diseases and also in cadmium intoxication.

**Disclosure:**

No significant relationships.

